# Vapocoolant Anesthesia for Cosmetic Facial Rejuvenation Injections: A Randomized, Prospective, Split-Face Trial

**Published:** 2018-02-07

**Authors:** Matthew R. Zeiderman, Shahrooz Sean Kelishadi, John Paul Tutela, Arun Rao, Saeed Chowdhry, Ronald M. Brooks, Bradon J. Wilhelmi

**Affiliations:** ^a^Division of Plastic & Reconstructive Surgery, Department of Surgery, University of Louisville School of Medicine, Louisville, Ky; ^b^SSK Plastic and Reconstructive Surgery, Newport Beach, Calif

**Keywords:** injections, pain, facial rejuvenation, neurotoxin, filler

## Abstract

**Background:** Minimally invasive cosmetic procedures are the most commonly performed aesthetic techniques by plastic surgeons. Patients are interested in a pain-free experience. Surgeons desire patient satisfaction and time-efficient utilization of office staff and resources. Clinical evidence exists for use of vapocoolant technology to reduce pain associated with intravenous cannulation in the pediatric population and in hemodialysis patients. Applying vapocoolant technology to facial rejuvenation is a novel approach to decrease pain associated with neurotoxin or filler injection. **Methods:** A randomized, prospective study was conducted, testing 15 subjects receiving filler injections and another 15 patients receiving neurotoxin injections using a split-face model. The vapocoolant spray used was composed of a 95:5 ratio of 1,1,1,3,3-pentafluoropropane and 1,1,1,2-tetrafluoroethane. Within each group, individual patients randomly received injection (filler or neurotoxin) alone versus injection (filler or neurotoxin) plus vapocoolant on an equivalent half of his or her face. An independent examiner recorded from each patient on a scale of 1 to 10 perceived pain for injection alone versus injection plus vapocoolant spray. Results were calculated as a percentage change of pain scores experienced after injection for each person between the control (nonvapocoolant) and treatment (vapocoolant) sides of the face. **Results:** Vapocoolant spray at the time of cosmetic facial injections leads to a 59% decrease in perceived pain score with neurotoxin injections (range, 0%-100% change) and 64% decrease in perceived pain score with filler injections (range, 0%-100% change). These results were statistically significant with *P* < .05. **Conclusion:** Vapocoolant spray reduces pain associated with facial rejuvenation procedures.

Minimally invasive cosmetic procedures are the most commonly performed aesthetic techniques by plastic surgeons and continue to increase in popularity. In 2016, 4.6 million botulinum toxin type A neurotoxin (Botox, Dysport, Xeomin) and 2.5 million soft-tissue dermal filler (eg, hyaluronic acid, polylactic acid, calcium hydroxylapatite) injection procedures were performed.^1^

Patients desire a pain-free experience. The anticipated and procedural pain of injection of neurotoxin and dermal fillers is of concern and anxiety to patients.^2^^,^^3^ Surgeons desire patient satisfaction and time-efficient utilization of office staff and resources. Many anesthetic modalities have been utilized to decrease the pain associated with facial rejuvenation injections, including ice, vibration, local anesthetics, and ointments.^3^^-^6 Attention has turned to the use of vapocoolant spray technology to decrease injection pain. Clinical evidence exists for use of vapocoolant technology to reduce pain associated with intravenous cannulation and injections for pediatric, adult, and hemodialysis patients.^7^^-^13 The findings in the pediatric population are particularly promising, as this age group tends to be more anxious and sensitive to painful stimuli. The adaptation of vapocoolant technology to facial rejuvenation is an alternative approach to decrease pain associated with neurotoxin or filler injection. This application may decrease anxiety about future treatments and improve patient satisfaction.

Few studies have explored the potential of vapocoolant spray for facial neurotoxin and filler injection analgesia. To date, 2 split-face control studies have been published analyzing the effectiveness of vapocoolant spray for botulinum toxin type A injections. A 2009 nonblinded study of 52 patients by Weiss and Lavin^14^ demonstrated that vapocoolant spray versus no anesthesia prior to glabellar neurotoxin injections decreased procedural pain and anxiety about future injections. A similar study by Engel and colleagues^15^ demonstrated vapocoolant spray to be effective analgesia for neurotoxin corrugator muscle injection in 20 patients. No studies have analyzed the effectiveness of vapocoolant spray as analgesia for dermal filler injections, which are generally considered to be more painful.

We present the first randomized, prospective, split-face model study to analyze the efficacy of vapocoolant spray for decreasing the pain of both neurotoxin and dermal filler facial injections.

## METHODS

**Figure 1:**
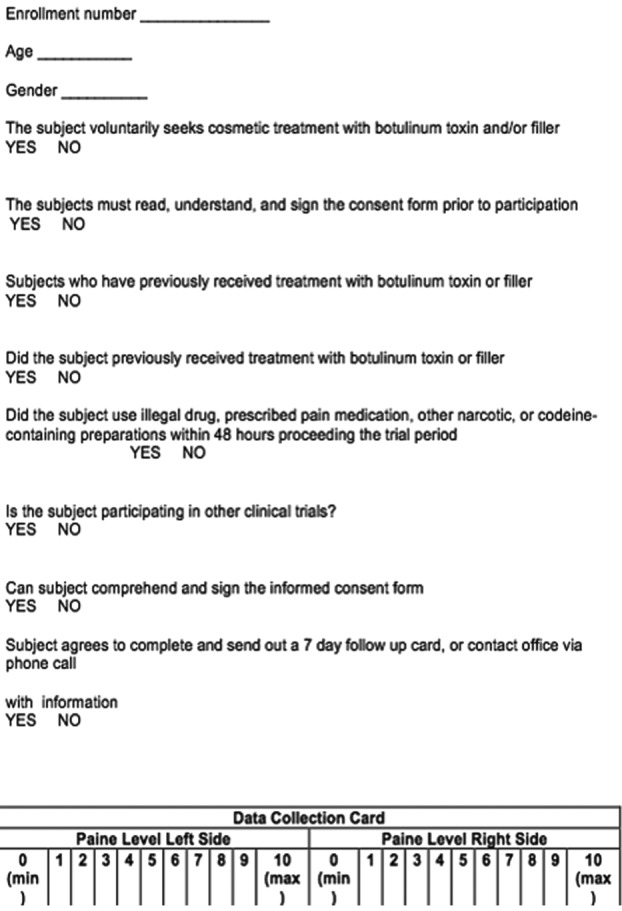
Data collection sheet.

Institutional review board approval was obtained from the University of Louisville School of Medicine. Male and female English-speaking literate volunteers aged 22 to 66 years who were either naive to or had prior exposure to neurotoxin or filler injections were studied. Subjects enrolled in other clinical studies or having consumed any narcotic medications within 48 hours of participation were excluded. The data collection sheet is demonstrated in Figure 1.

A randomized, prospective study was conducted testing 30 total people, with 15 patients receiving filler injections and another 15 patients receiving neurotoxin injections using a split-face model. The vapocoolant spray used was composed of a 95:5 ratio of 1,1,1,3,3-pentafluoropropane and 1,1,1,2-tetrafluoroethane. Within each group, subjects received in random order either the injectable corresponding to their group alone as a control or the injectable used in conjunction with the vapocoolant spray. Therefore, each patient randomly received injection (filler or neurotoxin) alone versus injection (filler or neurotoxin) plus vapocoolant on an equivalent half of his or her face. An independent examiner recorded from each patient on a pain scale of 1 to 10 (the higher the number, the greater the perceived pain) for injection alone versus injection plus vapocoolant spray. The results were then calculated as a percentage change of pain scores experienced after injection for each person between the control (nonvapocoolant) and vapocoolant sides of the face. Statistical significance was calculated using the paired 2-tailed *t* test.

## RESULTS

Average patient age for filler was 50.33 years (SD = 8.13; range, 27-66 years) and for neurotoxin was 39.53 years (SD = 11.31; range, 26-66 years). Six of 15 patients receiving fillers were filler naive. Seven of 15 receiving neurotoxin were neurotoxin naive.

The average pain score for neurotoxin injections decreased from 3.37 without vapocoolant spray to 1.47 with spray (*P* = .0002). The average pain score for filler injection decreased from 4.3 without spray to 1.57 with spray (*P* = .00006). Nine of 15 patients receiving neurotoxin treatment reported pain scores of 0 to 1 with vapocoolant spray. Eight of 15 patients receiving dermal filler reported pain scores of 0 to 1 with vapocoolant spray (Tables 1 and 2).

**Figure 2:**
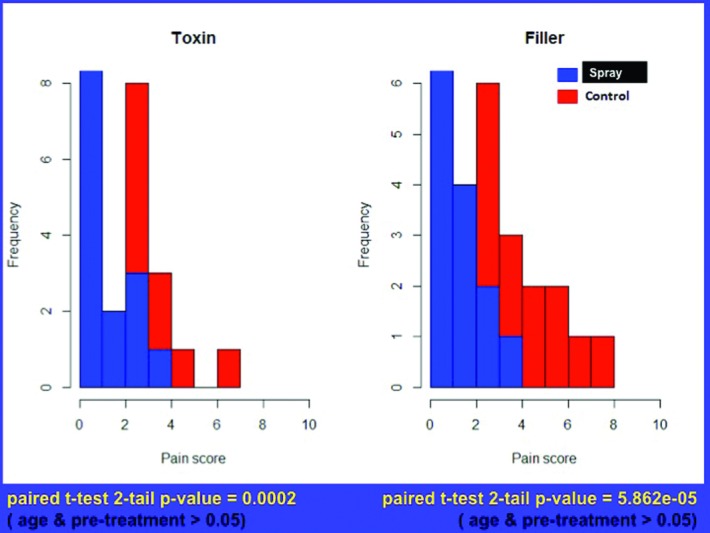
A left shift is depicted in pain score (decreased pain) for both neurotoxin (on the left) and filler (on the right) injections with the addition of vapocoolant spray. Scores in both groups were consistently higher without vapocoolant spray as shown in the red bars toward the right of each scale. Statistical significance was shown between the control and treatment groups for both neurotoxin and filler injections. Having had prior injection experience did not show statistical significance in pain scores when compared with injection-naive subjects.

Vapocoolant spray at the time of cosmetic facial injections leads to a 59% decrease in perceived pain score with neurotoxin injections (range, 0%-100% change) and 64% decrease in perceived pain score with filler injections (range, 0%-100% change). These results were statistically significant at *P* < .05 (Fig 2).

## DISCUSSION

Maximizing patient comfort is important to patient satisfaction with all procedures. This is particularly true with elective cosmetic procedures. To provide patient satisfaction with cosmetic injections, several patient values must be considered: the ability to fit treatments into a busy schedule; instant pain relief; and reduced anxiety over injections. The use of an efficacious, cost-effective, fast-acting analgesic technique is thus essential to deliver pain-free facial rejuvenation. Vapocoolant spray is ideal because it is quick and cost-effective, provides adequate pain relief, and yields patient satisfaction. This allows surgeons the efficiency and capacity to treat more patients, utilize cutting-edge technology to benefit patient care, reallocate clinical staff for other priorities, and spend more time on more profitable clinical procedures.

Each form of local anesthesia has inherent benefits and disadvantages. Vapocoolant is a compressed liquid form of halogenated alkanes that upon decompression and exposure to the warm skin environment changes to vapor. Upon skin contact, the phase change from liquid to vapor causes an endothermic reaction that cools the skin. The drop in temperature decreases the conduction velocity of A-δ and C nerve fibers and dampens pain signals.^8^^,^^16^ This is similar to the mechanism of action of ice, which also decreases conduction velocity of nociceptive nerve fibers. Vapocoolant can be administered simultaneously with injection, thereby providing instant pain relief for the comfort. At a cost of less than $1 per dose, vapocoolant is very cost-effective.^15^^,^^17^ This is particularly important for reducing the cost of patient care when a treatment with 50 units of neurotoxin costs roughly $600 and the leading hyaluronic acid dermal fillers have an average cost of $591.

Ice is cheap, readily available, and easy to use. Its analgesic and vasoconstrictive properties have been known for centuries, and multiple studies have analyzed its effectiveness for neurotoxin injection analgesia.^18^^-^20 However, the use of ice or ice packs is cumbersome, is not always effective, and predisposes to cold burns and uneven analgesia.

EMLA, or eutectic mixture of local anesthetics, is a topical anesthetic cream composed of 2.5% lidocaine and 2.5% prilocaine that is demonstrated to be an effective analgesic for neurotoxin injections.^18^^,^^20^^,^^21^ However, it requires 30 to 60 minutes to achieve maximal effect and costs about $15.00 for a 2-dose tube.^9^^,^^14^ This requires patients to apply the cream before coming in for treatment or sit in the office awaiting treatment after application, which is undesirable for those with busy schedules. A recent study by Cohen and colleagues^22^ demonstrated effective pain relief with a topical anesthetic cream composed of 7% lidocaine and 7% tetracaine (Pliaglis) for dermal hyaluronic acid fillers in a placebo-controlled, double-blinded study that enrolled 70 patients. However, like other topical analgesics, this cream requires at least 20 to 30 minutes after application to be effective and 60 minutes for maximal efficacy.^23^^,^^24^

Vibration therapy has been shown to reduce pain for cosmetic facial dermal filler and neurotoxin injections.^4^^,^^5^ This method reduces but does not eliminate pain by creating concurrent non-noxious stimuli that decrease perceived pain intensity.^5^ There do not appear to be adverse side effects from this technique, but this method requires an assistant or single-handed injection by the physician. Furthermore, vibration may interfere with needle placement and the dermal filler or neurotoxin injection process. Since vibration alone reduces but does not eliminate pain, another form of topical or local anesthetic in conjunction may be necessary to ensure patient comfort.

Other forms of anesthesia commonly used for dermal fillers includes spot contact cooling systems, local anesthetic, and the combination of anesthetic with filler.^3^^,^^6^ Local anesthetics effectively decrease pain but cause tissue distortion at the injection site, and improper injection carries a risk of allergic reaction, irregular heartbeat, and seizure.^3^^,^^25^ When improperly applied, topical anesthetics carry a similar risk and may cause skin irritation.^3^ The inclusion of anesthetic in the filler preparation will not provide anesthesia at the time of needle insertion but may provide temporary relief from symptoms of tissue expansion and edema upon filler injection.

The application of vapocoolant spray is safe and local and provides instant relief from anxiety and pain of injection. Our study is the first to look at vapocoolant spray for pain control for both neurotoxin and dermal filler cosmetic injections. Using a split-face model, each patient randomly received injection (filler or neurotoxin) alone versus injection (filler or neurotoxin) plus vapocoolant on an equivalent half of his or her face and a blinded investigator collected the data of the patient's perceived pain score.

Pain is a subjective experience. Consequently, truly objective data cannot be obtained for perceived pain scores. It is not possible to control for how anticipated pain and anxiety may affect reported pain scores. Individuals may over- or underestimate their perceived pain. Although the patient feels which side receives vapocoolant spray, a placebo-like effect may influence the results. However, the tendency for individuals to over- or underestimate pain is likely balanced out in the aggregate analysis. Because it is difficult to standardize perceived pain between individuals, the individual pain scores were converted to the percentage change of perceived pain for each individual to further balance out the variability among subjects. Our aggregate analysis showed statistically significant decreases in pain of 59% and 64% for neurotoxin and dermal filler injections, respectively, when patients were treated with vapocoolant spray. There was no statistical difference in pain scores between patients who were naive to facial injection procedures and those who had prior experience(s). Our results demonstrate the vapocoolant anesthesia spray to provide statistically significant improvements in pain control during neurotoxin and dermal filler cosmetic injections.

## CONCLUSIONS

We present the first randomized, prospective, split-face study to analyze vapocoolant spray for pain control for both neurotoxin and dermal filler cosmetic injections. Our results demonstrate vapocoolant spray to provide statistically significant pain reduction for facial rejuvenation injections. Vapocoolant spray overcomes the shortcoming of other local anesthetic methods, as it is easy to use, acts instantly, does not distort the injection site, and is cost-effective. The use of an efficacious, cost-effective, fast-acting analgesic technique is essential to deliver pain-free facial rejuvenation. Our study demonstrates vapocoolant spray to meet these criteria to provide optimal pain relief and patient satisfaction for neurotoxin and dermal filler facial rejuvenation injections.

## Figures and Tables

**Table 1:** Neurotoxin results*

Age, y	Without spray	With spray	% change	Had toxin before
66	3	0.5	−83	Yes
53	3	1	−67	No
30	4	3	−25	Yes
39	2.5	2.5	0	Yes
41	1	0	−100	Yes
47	4	4	0	No
27	7	1	−86	Yes
31	3	2	−33	Yes
33	2	1	−50	Yes
26	3	0	−100	No
47	3	0	−100	Yes
28	3	1	−67	No
48	3	2	−33	No
43	4	1	−75	No
34	5	3	−40	No
Average	3.37	1.47	−59	

*Neurotoxin injection pain scores of individual subjects are shown without any analgesia and also with the addition of vapocoolant spray. The % change in pain score perceived is also calculated between no treatment and the addition of vapocoolant treatment. The average scores of the whole group are also shown in the bottom row for controls, addition of vapocoolant spray, and the % change in pain score.

**Table 2:** Filler results*

Age, y	Without spray	With spray	% change	Had filler before
66	3	1	−67	Yes
54	3	2	−33	No
52	3	1	−67	No
58	2	2	0	No
43	7	0	−100	Yes
42	6	1	−83	Yes
47	3.5	1.5	−57	No
44	2	1	−50	Yes
47	5	1	−80	Yes
47	8	3	−63	Yes
54	6	4	−33	No
63	4	2	−50	Yes
57	4	0	−100	Yes
40	3	1	−67	No
41	5	3	−40	Yes
Average	4.3	1.57	−64	

*Filler injection pain scores of individual subjects are shown without any analgesia and with the addition of vapocoolant spray. The % change in pain score perceived is also calculated between no treatment and the addition of vapocoolant treatment. The average scores of the whole group are also shown in the bottom row for controls, addition of vapocoolant spray, and the % change in pain score.
